# Cholesterol-crystalized coronary atheroma as a potential precursor lesion causing acute coronary syndrome: a case report

**DOI:** 10.1093/ehjcr/ytz128

**Published:** 2019-07-16

**Authors:** Hiroki Sugane, Yu Kataoka, Fumiyuki Otsuka, Satoshi Yasuda

**Affiliations:** 1 Department of Cardiovascular Medicine, National Cerebral and Cardiovascular Center, 5-7-1 Fujishirodai, Suita, Osaka, Japan; 2 Department of Cardiovascular Medicine, Chikamori Hospital, 1-1-16 Okawasuji, Kochi, Japan

**Keywords:** Cholesterol crystal, Plaque progression, Optical coherence tomography, Near-infrared spectroscopy, Coronary artery disease, Case report

## Abstract

**Background:**

Histopathological studies have reported the presence of cholesterol crystals in the culprit lesion in patients with sudden cardiac death. Given that cholesterol crystals themselves promote pro-inflammatory cascades, they may destabilize atherosclerotic plaques, leading to the occurrence of acute coronary events.

**Case summary:**

A 60-year-old man presented with ST-segment elevation myocardial infarction. Emergent coronary angiography revealed a severely stenotic lesion (=culprit lesion) and another non-obstructive lesion in the proximal and middle segments of the left anterior descending artery (LAD), respectively. Optical coherence tomography (OCT) imaging showed that both lesions exhibited lipid-rich plaque with cholesterol crystals, and the non-obstructive lesion in the mid-LAD did not have a thin fibrous cap (its thickness = 230 μm). A drug-eluting stent was successfully implanted at the culprit lesion in the proximal LAD. On non-contrast T1-weighted magnetic resonance imaging performed 10 days after percutaneous coronary intervention (PCI), a high-intensity signal was identified at the non-obstructive mid-LAD lesion. This lesion was medically managed with aspirin, clopidogrel, and rosuvastatin due to the absence of myocardial ischaemia. However, 12 months after PCI, the patient was hospitalized again due to unstable angina pectoris. Coronary angiography revealed substantial progression of the mid-LAD lesion. Optical coherence tomography imaging prior to the second PCI showed a severely narrowed lesion containing cholesterol crystals and covered by organized thrombus. This lesion harbored an extensive amount of lipidic materials on near-infrared spectroscopy (maximum 4-mm lipid core burden index = 809).

**Discussion:**

In our case, atherosclerotic plaques containing cholesterol crystals was associated with the occurrence of acute coronary syndrome. Our findings suggest that plaque with cholesterol crystals is a potential precursor to future acute coronary events.

**Learning points**

- Evaluation of not only fibrous cap thickness but also coronary plaque morphology including cholesterol crystal may enable to predict the occurrence of acute coronary syndrome (ACS).
- Randomized control trials will be required to investigate whether additional therapeutic approach modulating cholesterol crystal could prevent future ACS events.

## Introduction

Cholesterol crystal is a morphological feature of atherosclerotic plaques and is frequently observed at the culprit lesions of acute coronary syndrome (ACS).^1^ These crystals form through the accumulation of excessive amount of cholesterol materials within vessel walls. Biologically, the formation of cholesterol crystals activates the nucleotide-binding domain and leucine-rich repeat protein-3 inflammasome and then stimulates the secretion of interleukin-1β, which promotes atherogenesis.^2^^,^^3^ This observation suggests that cholesterol crystal may be morphological features of vulnerable plaques due to its pro-inflammatory property and needle-like structure.

Optical coherence tomography (OCT) imaging enables the visualization of cholesterol crystals within vessel walls *in vivo*.^4^ Coronary atheroma containing cholesterol crystal is characterized by the presence of lipid-rich plaque.^5^ This clinical finding also indicates that the formation of cholesterol crystals may promote plaque instability. However, whether or not cholesterol crystal formation is associated with future coronary events remains to be determined yet. Here, we report a case in which ACS occurred repeatedly due to plaques containing cholesterol crystals.

## Timeline

**Table:** 

Time points	Events
2016 November	Severe, prolonged (6-h) chest pain. Electrocardiogram revealed anterior wall myocardial infarction with ST-segment elevation. Coronary angiography showed one obstructive and another non-obstructive stenotic lesion in the left anterior descending artery (LAD). The obstructive lesion was treated with a drug-eluting stent. The non-obstructive lesion harbored cholesterol crystals and had a thick fibrous cap, as demonstrated by optical coherence tomography imaging.
2016 December	Non-contrast T1-weighted magnetic resonance imaging showed a high-intensity signal corresponding to the non-obstructive lesion containing cholesterol crystals.
	Patient discharged on standard medical therapy, including dual antiplatelets and rosuvastatin.
2017 December	Recurrence of chest pain at rest.
	Coronary angiography showed substantial progression of the non-obstructive lesion containing cholesterol crystals. Optical coherence tomography imaging showed severe LAD narrowing at the lesion site due to overlying tissues that presumably consisted of thrombus. Near-infrared spectroscopy imaging visualized a considerable amount of lipidic materials at the lesion, reflected by a maximum 4-mm lipid core burden index of 809. Drug-eluting stent successfully implanted.
	Patient discharged with further lipid-lowering therapy with ezetimibe.
2018 December	Last follow-up; uneventful clinical course.

## Case presentation

**Figure 1:**
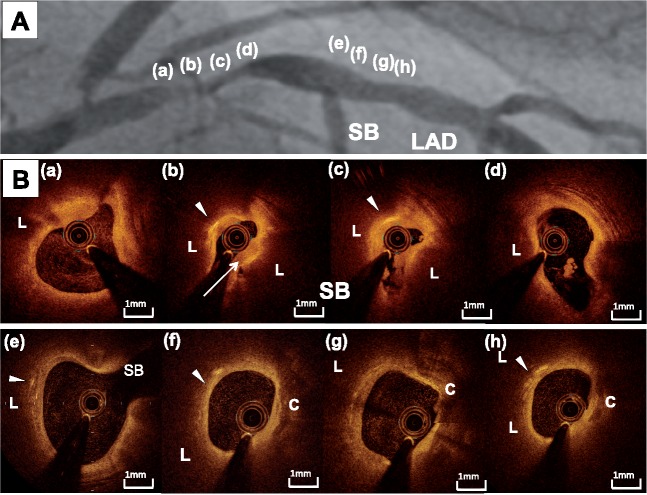
(*A*) Coronary angiography prior to percutaneous coronary intervention. One severe stenosis (a–d) and another non-obstructive lesion (e–h) were identified in the proximal and middle segments of left anterior descending artery, respectively. (a–h) corresponds to optical coherence tomography images in (*B*). (*B*) Optical coherence tomography Images prior to percutaneous coronary intervention. Culprit lesion (a–d) exhibited lipid-rich plaque (L) with cholesterol crystal (arrow head). Homogeneous high-intensity signal area at the surface of this lesion was observed [arrow in (b)]. Fibrous cap thickness was 110 μm. At another non-obstructive lesion (e–h), lipid-rich plaque (L), multiple cholesterol crystals (arrow head), and small calcification (C) were visualized. The thickness of its fibrous cap was 230 μm. SB, septal branch.

**Figure 2:**
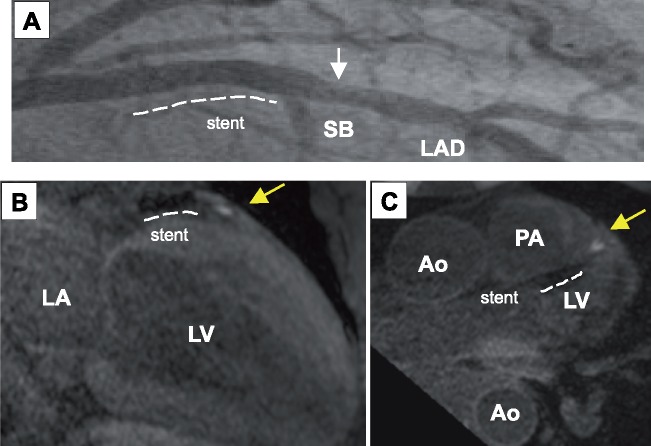
(*A*) Coronary angiography after percutaneous coronary intervention. Zotarolimus-eluting stent [dashed line: (3.5 × 18 mm Resolute Integrity^®^, Medtronic, Minneapolis, MN, USA)] was successfully implanted at culprit lesion. The non-obstructive lesion with cholesterol crystal (arrow) was medically managed. (*B*) Non-contrast T1-weighted magnetic resonance imaging after percutaneous coronary intervention (sagittal view). (*C*) Non-contrast T1-weighted magnetic resonance imaging after percutaneous coronary intervention (coronal view). High-intensity signal (yellow arrow) was observed at the coronary lesion distal to the implanted stent (dashed line), which corresponded to the non-obstructive plaque in left anterior descending artery. Ao, aorta; LA, left atrium; LV, left ventricle; PA, pulmonary artery; SB, septal branch.

A 60-year-old man with a history of dyslipidaemia presented to our hospital due to 6 h of central chest pain at rest, with radiation into his left shoulder. He had not previously received any medication for dyslipidaemia. His vital signs were stable, and he had no cardiac murmurs, jugular vein distension, or pitting oedema (heart rate, 81 beats per minute; blood pressure, 169/109 mmHg; respiratory rate, 16/min; oxygen saturation, 98% on ambient room air). Electrocardiogram showed ST-segment elevation in the anterior leads (V_2_–V_6_), with reciprocal changes in the inferior leads (II, III, aVf). Transthoracic echocardiography identified severe hypokinesis of the left ventricular anterior wall (left ventricular ejection fraction, 40%). Emergent coronary angiography revealed a severely stenotic lesion (=culprit lesion) and another non-obstructive lesion in the proximal and middle segments of the left anterior descending artery (LAD), respectively (*Figure 1A*, Supplementary material online, *Video S1*). Optical coherence tomography imaging (Dragonfly OPTIS^TM^; Abbott, Chicago, IL, USA) was conducted after manual thrombus aspiration to evaluate the lesions. The culprit lesion exhibited lipid-rich plaques that contained multiple cholesterol crystals and caused severe narrowing (*Figure 1B*, Supplementary material online, *Video S2*). The surface of the culprit lesion was covered by homogeneous tissue that was considered to be a thrombus. The non-obstructive lesion in the middle segment of the LAD also contained cholesterol crystals and had a thick fibrous cap (thickness, 230 μm) (*Figure 1C*, Supplementary material online, *Video S2*). One 3.5 mm × 18 mm Resolute Integrity^®^ zotarolimus-eluting stent (Medtronic, Minneapolis, MN, USA) was successfully implanted at the culprit lesion under OCT guidance (*Figure 2D*, Supplementary material online, *Video S3*). Since the patient had a high level of low-density lipoprotein cholesterol (LDL-C) on admission (LDL-C, 4.1 mmol/L; total cholesterol, 6.3 mmol/L; triglyceride, 0.7 mmol/L; high-density lipoprotein cholesterol (HDL-C), 1.8 mmol/L; glycated haemoglobin, 5.0%), rosuvastatin 10 mg was commenced, in addition to aspirin 100 mg, clopidogrel 75 mg, enalapril 2.5 mg, and carvedilol 10 mg. Non-contrast T1-weighted magnetic resonance imaging (T1WI-MRI) 10 days after PCI showed a high-intensity signal distal to the implanted stent, corresponding to the non-obstructive lesion containing cholesterol crystals that was visualized by OCT (*Figure 2E* and *F*, Supplementary material online, *Video S4*). Since a stress myocardial perfusion scan did not show any residual myocardial ischaemia, this lesion was medically managed.

**Figure 3:**
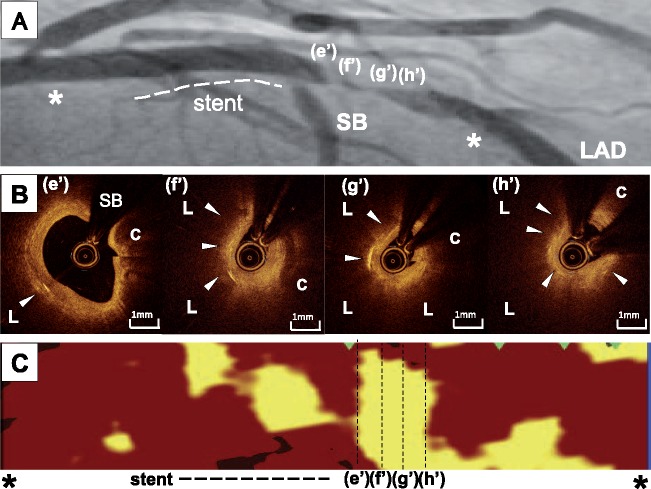
(*A*) Coronary angiography 12 months after the first percutaneous coronary intervention. While in-stent restenosis did not occur within the previously implanted stent (dashed line), progression of the non-obstructive plaque was observed (e′–h′). (e′–h′) corresponds to optical coherence tomography images in (*B*) and near-infrared spectroscopy images in (*C*). Two asterisks (*) shows the evaluated segment on near-infrared spectroscopy imaging (*C*). (*B*) Optical coherence tomography imaging prior to the second percutaneous coronary intervention. Cholesterol-crystalized lesion (arrow head) was severely narrowed due to large lipid-rich plaque (L) and its overlying tissues presumably consisting of thrombus (arrow). (*C*) Near-infrared spectroscopy imaging prior to the second percutaneous coronary intervention. Near-infrared spectroscopy imaging visualized the presence of extensive amount of lipidic materials at the progressive cholesterol-crystalized lesion (maximum 4-mm lipid core burden index = 809). C, calcification; LAD, left anterior descending artery; SB, septal branch.

Twelve months later, the patient was hospitalized again due to 3 days of intermittent central chest pain at rest. There were no abnormalities on physical examination (heart rate, 63 b.p.m.; blood pressure, 115/61 mmHg; respiratory rate, 14/min; oxygen saturation, 97% on ambient room air). The patient was still taking dual-antiplatelet therapy (aspirin 100 mg and clopidogrel 75 mg) with 10 mg rosuvastatin, 2.5 mg enalapril, and 10 mg carvedilol (LDL-C, 2.2 mmol/L; total cholesterol, 4.6 mmol/L; triglyceride, 0.9 mmol/L; HDL-C, 1.9 mmol/L; glycated haemoglobin, 5.3%). Despite a normal troponin T level (0.01 ng/mL) and no ST-T changes on electrocardiogram or new regional left ventricular wall motion abnormality on echocardiogram, coronary angiography revealed substantial progression of the non-obstructive lesion containing cholesterol crystals, whereas the implanted zotarolimus-eluting stent did not show restenosis (*Figure 3G*, Supplementary material online, *Video S5*). Optical coherence tomography and near-infrared spectroscopy (NIRS) imaging were used to evaluate the culprit lesion prior to PCI. On OCT imaging, this lesion was severely narrowed due to the overlying tissue that presumably consisted of thrombus (*Figure 3H*, Supplementary material online, *Video S6*). In addition, a considerable amount of lipidic material at this lesion was visualized by NIRS imaging [maximum 4-mm lipid core burden index (LCBI) = 809] (*Figure 3I*). An additional 3.5 mm × 24 mm Synergy^®^ everolimus-eluting stent (Boston Scientific, Marlborough, MA, USA) was successfully implanted. Since an increased dose of rosuvastatin would only lower LDL-C levels by ∼6%, 10 mg ezetimibe was added instead to achieve the Japanese target LDL-C level (<1.8 mmol/L). The patient’s course was uneventful in the 12 months after the second PCI. Her LDL-C level under rosuvastatin and ezetimibe was 1.6 mmol/L.

## Discussion

Vulnerable plaque, considered to be an important driver of acute coronary events, has been defined by histopathological studies as a lesion with a thin fibrous cap overlying a large necrotic core. In our case, however, ACS occurred due to the rapid progression of the lesion with associated cholesterol crystallization and a thick fibrous cap. This observation suggests that plaque containing cholesterol crystals may be another precursor lesion associated with ACS.

Increased cholesterol content within vessel walls affects lipid metabolism in macrophages, leading to the deposition of cholesterol crystals within plaques.^1–3^ Cholesterol crystals have been shown to directly stimulate the production of interleukin-1β, suggesting their potential to destabilize plaques.^2^^,^^3^ In our case, the culprit lesion underlying the initial ST-segment elevation myocardial infarction episode contained cholesterol crystals in its overlying thrombus. Additionally, the second ACS event occurred due to rapid progression and thrombus formation at the other lesion with associated cholesterol crystallization. Given that interleukin-1βinduce plaque rupture and its thrombus formation via the increased expression of matrix metalloproteinase, tissue factor, and monocyte chemoattractant protein-1,^6^^,^^7^ cholesterol crystals may increase lesion vulnerability and increase the risk of acute coronary events.

T1-weighted magnetic resonance imaging and NIRS imaging provide mechanistic insights into the association of cholesterol crystals with ACS. In the current case, a high-intensity signal on T1WI-MRI was identified at the lesion that caused the second ACS event. One recent study using OCT imaging reported that coronary plaques with high-intensity signals on T1WI-MRI were associated with lipid-rich plaque and healed plaque rupture but not with a thin fibrous cap.^8^ Considering that coronary plaque characterized by a high-intensity signal is associated with future coronary events,^9^ progression of the high-intensity signal plaque in our case suggests that cholesterol crystals may be an important feature of precursor lesions that may induce coronary events. Near-infrared spectroscopy imaging is an intravascular plaque imaging modality that quantitatively measures lipidic materials within coronary atheromas *in vivo.*^10^ The NIRS-derived LCBI has been validated by several histopathological studies that compared NIRS images with coronary specimens.^10^^,^^11^ LCBI_max4mm_ >400 has been considered as the presence of a high-risk plaque containing lipidic materials.^10^ Since the accumulation of lipidic materials within the vessel wall is a driver of cholesterol crystal formation, very high LCBI_max4mm_ at rapidly progressive plaques containing cholesterol crystals may account for the further development of cholesterol crystals *in vivo*.

In our case, the coronary plaques associated with ACS events did not have thin fibrous caps. This is inconsistent with postmortem studies that demonstrated that fibrous caps with thicknesses <65 μm were a major feature of culprit lesions in patients with acute coronary events.^12^^,^^13^ A recent *in vivo* OCT imaging study reported that the rupture of fibrous caps did not necessarily occur at their thinnest site.^14^ Of note, the median thickness of the fibrous cap overlying most ruptured plaques was below 188 μm. These findings suggest that ACS occurs even if the fibrous cap at the coronary plaque is not thin. Imaging assessment not just of fibrous cap thickness but also of plaque microstructure, including cholesterol crystals, may be important to identify high-risk lesions causing ACS.

The current case indicates the need for preventive management in patients with plaques containing cholesterol crystals. While an *in vitro* study reported that a statin modulated the density of cholesterol crystals, recurrence of ACS occurred due to progression of lesions containing cholesterol crystals despite rosuvastatin use.^15^ Further investigation is warranted to determine if more potent lipid-lowering agents, such as proprotein convertase subtilisin/kexin type 9 inhibitors, affect cholesterol crystals. The canakinumab anti-inflammatory thrombosis outcomes (CANTOS) study demonstrated a significant reduction of atherosclerotic cardiovascular events by an anti-inflammatory agent that modifies interleukin-1β.^16^ Considering that cholesterol crystals themselves promote the secretion of interleukin-1β, this therapeutic approach may be another valid strategy to prevent coronary events associated with cholesterol crystals.

## Conclusions

In conclusion, coronary plaques characterized by the presence of cholesterol crystals and thick fibrous caps were associated with the occurrence of ACS. Our findings suggest that cholesterol crystals may be an important feature of ACS precursor lesions and may require additional anti-atherosclerotic management.

## Lead author biography

**Figure:**
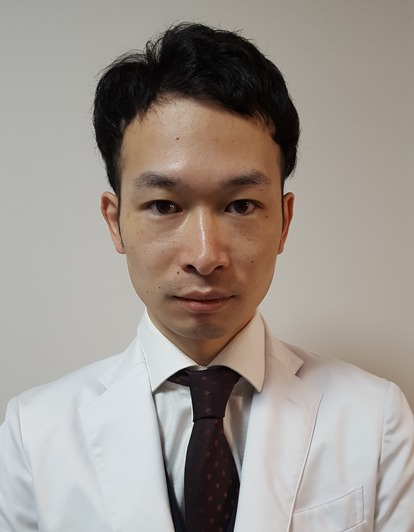


Hiroki Sugane was born in Chiba, Japan, in 1987. He graduated from Kochi Medical University and received the MD degree in 2012. He was working as a Cardiology fellow at Chikamori hospital from 2014. Then, he became a cardiovascular interventional fellow at Department of Cardiovascular Medicine, National Cerebral and Cardiovascular Center, Japan from 2017. He experienced a large number of PCI cases as well as analysis of intravascular imaging. From 2019, he serves as a staff cardiologist at Chikamori hospital, Kochi, Japan. His research interest is percutaneous coronary intervention and intravascular imaging.

## Supplementary Material

ytz128_Supplementary_Data

## Supplementary material

**Slide sets:** A fully edited slide set detailing this case and suitable for local presentation is available online as Supplementary data.

**Consent:** The author/s confirm that written consent for submission and publication of this case report including image(s) and associated text has been obtained from the patient in line with COPE guidance.
