# Supplementary material for: Cholesterol-crystalized coronary atheroma as a potential precursor lesion causing acute coronary syndrome: a case report
Source: Eur Heart J Case Rep. 2019 Jul 16;3(3):ytz128. doi: 10.1093/ehjcr/ytz128 (PMC6764557; doi:10.1093/ehjcr/ytz128)
Supplement: ytz128_Supplementary_Data [file ytz128_supplementary_data.zip › ytz128-Suppl_data/Supplementary_Slide_Set.pptx]

## Slide 1
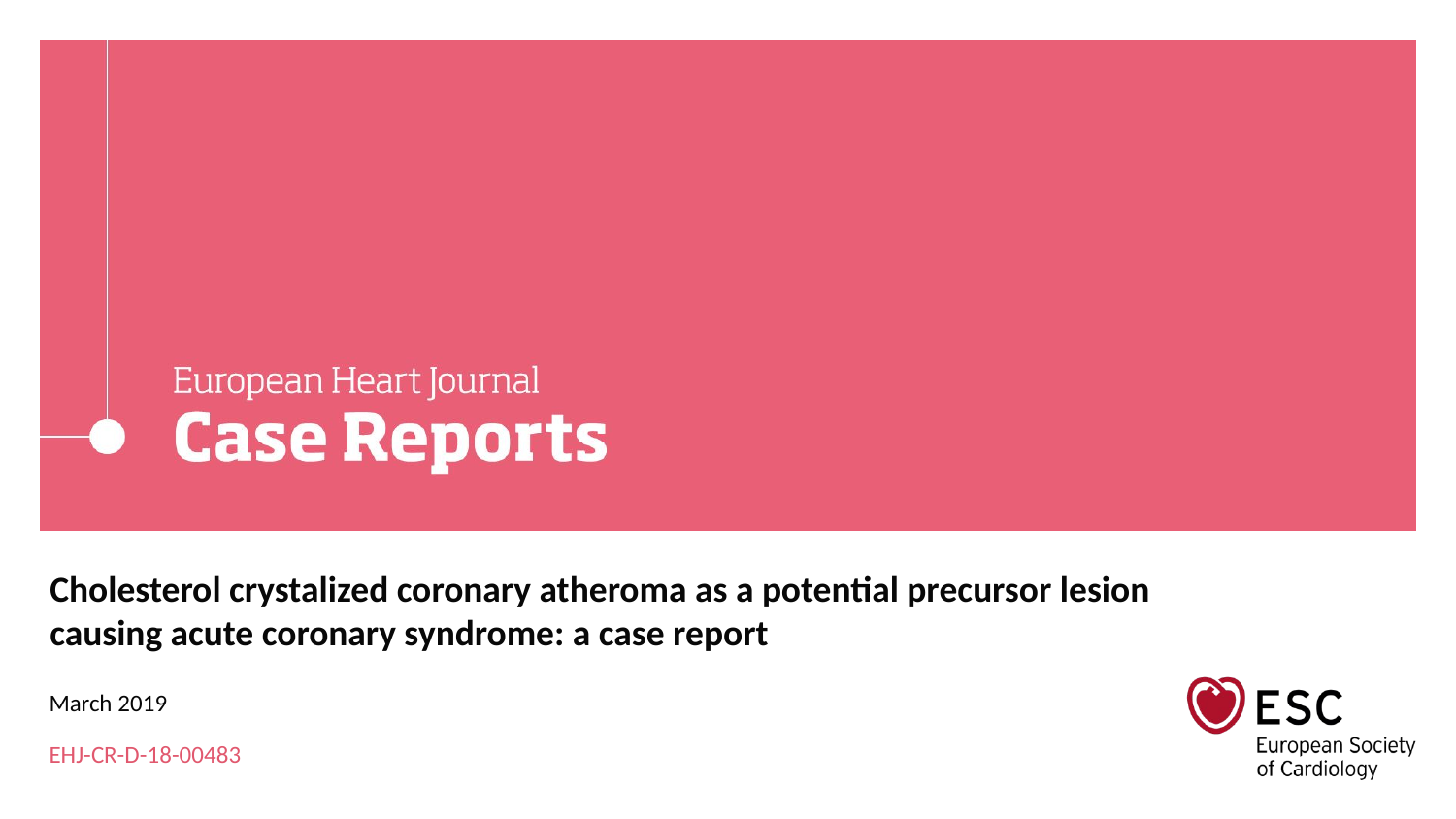

# Cholesterol crystalized coronary atheroma as a potential precursor lesion causing acute coronary syndrome: a case report
March 2019
EHJ-CR-D-18-00483

## Slide 2
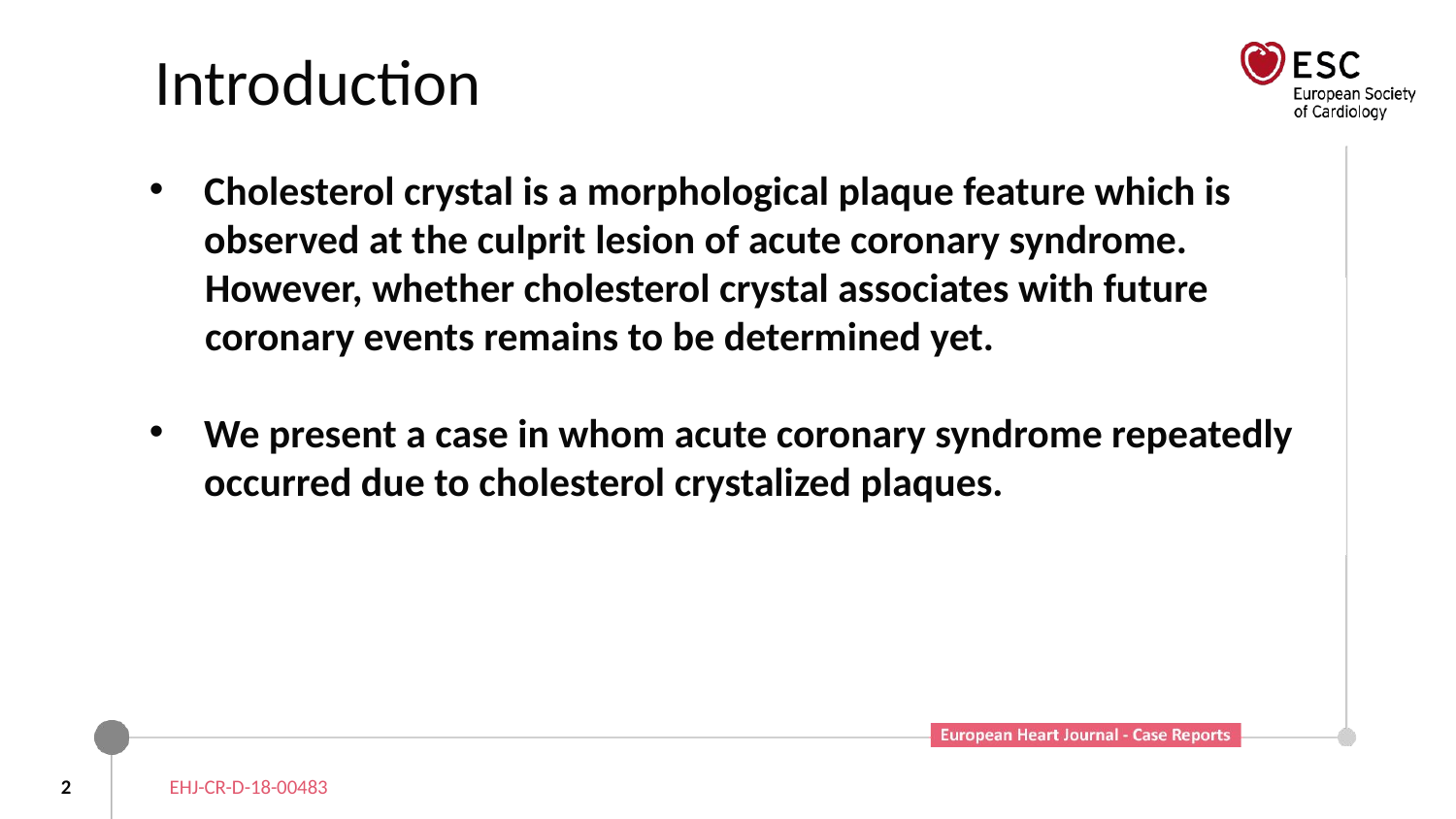

# Introduction
Cholesterol crystal is a morphological plaque feature which is observed at the culprit lesion of acute coronary syndrome.
 However, whether cholesterol crystal associates with future
 coronary events remains to be determined yet.
We present a case in whom acute coronary syndrome repeatedly occurred due to cholesterol crystalized plaques.
2
EHJ-CR-D-18-00483

## Slide 3
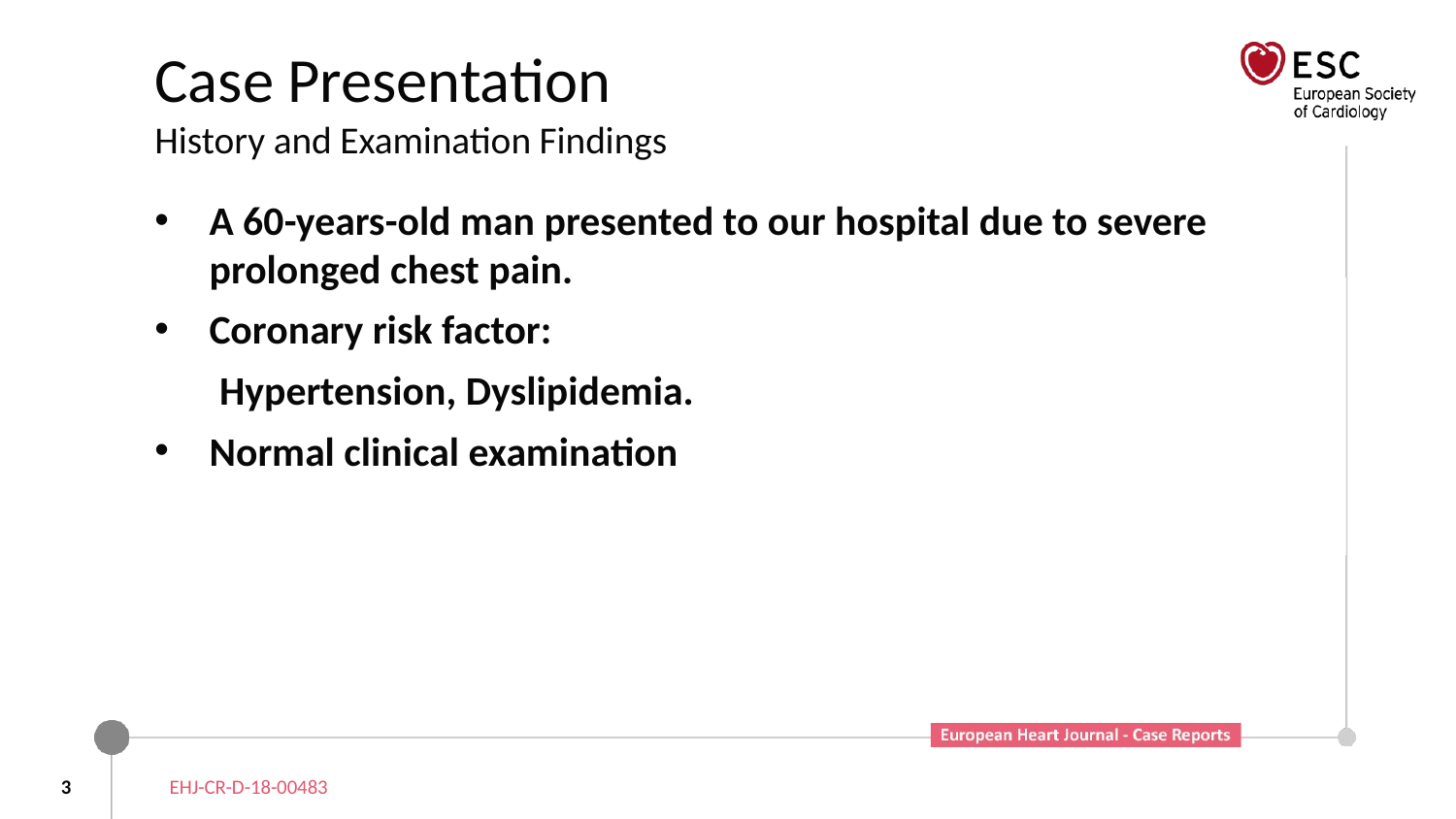

# Case Presentation History and Examination Findings
A 60-years-old man presented to our hospital due to severe prolonged chest pain.
Coronary risk factor:
 Hypertension, Dyslipidemia.
Normal clinical examination
3
EHJ-CR-D-18-00483

## Slide 4
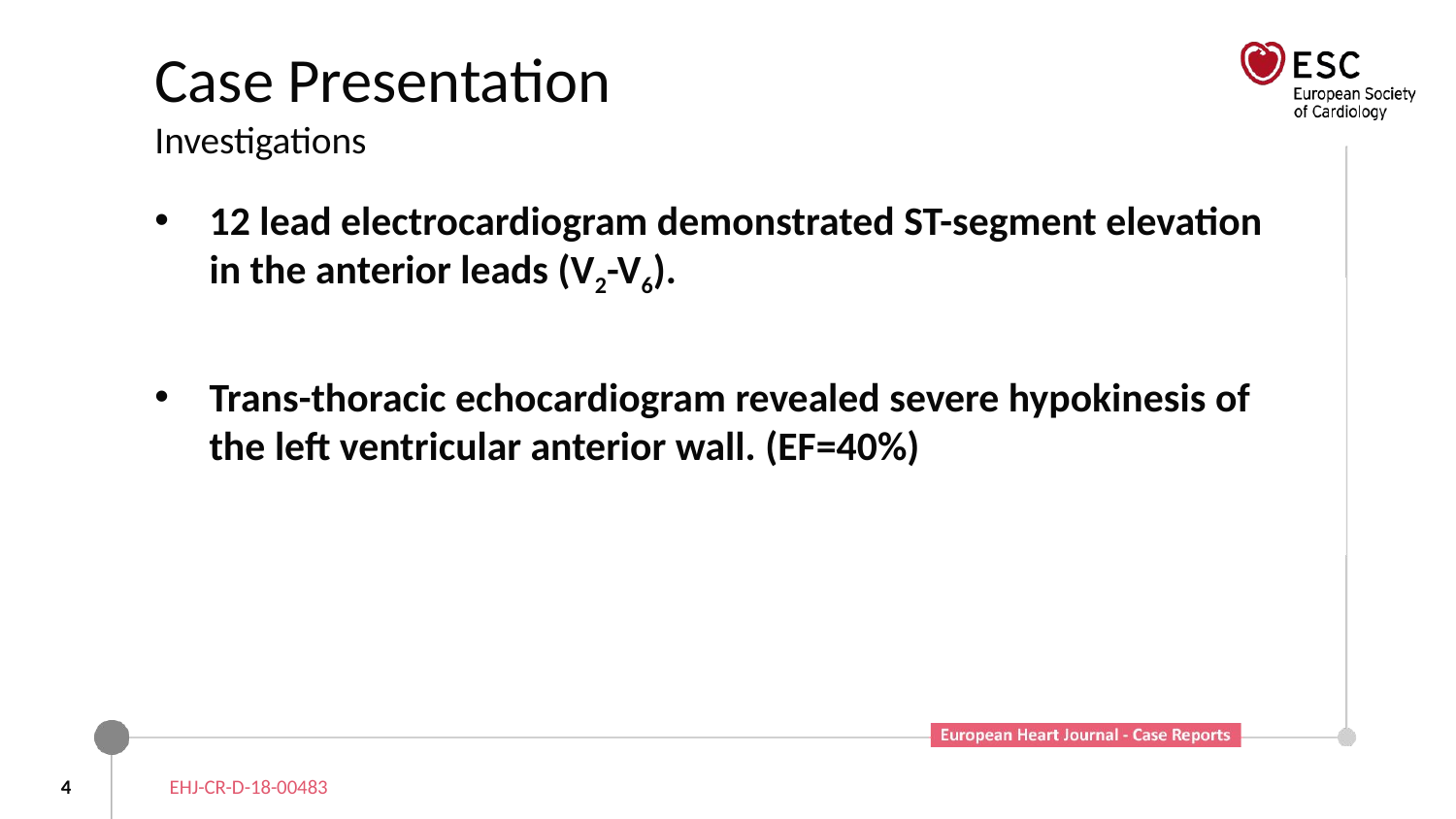

# Case Presentation Investigations
12 lead electrocardiogram demonstrated ST-segment elevation in the anterior leads (V2-V6).
Trans-thoracic echocardiogram revealed severe hypokinesis of the left ventricular anterior wall. (EF=40%)
4
EHJ-CR-D-18-00483

## Slide 5
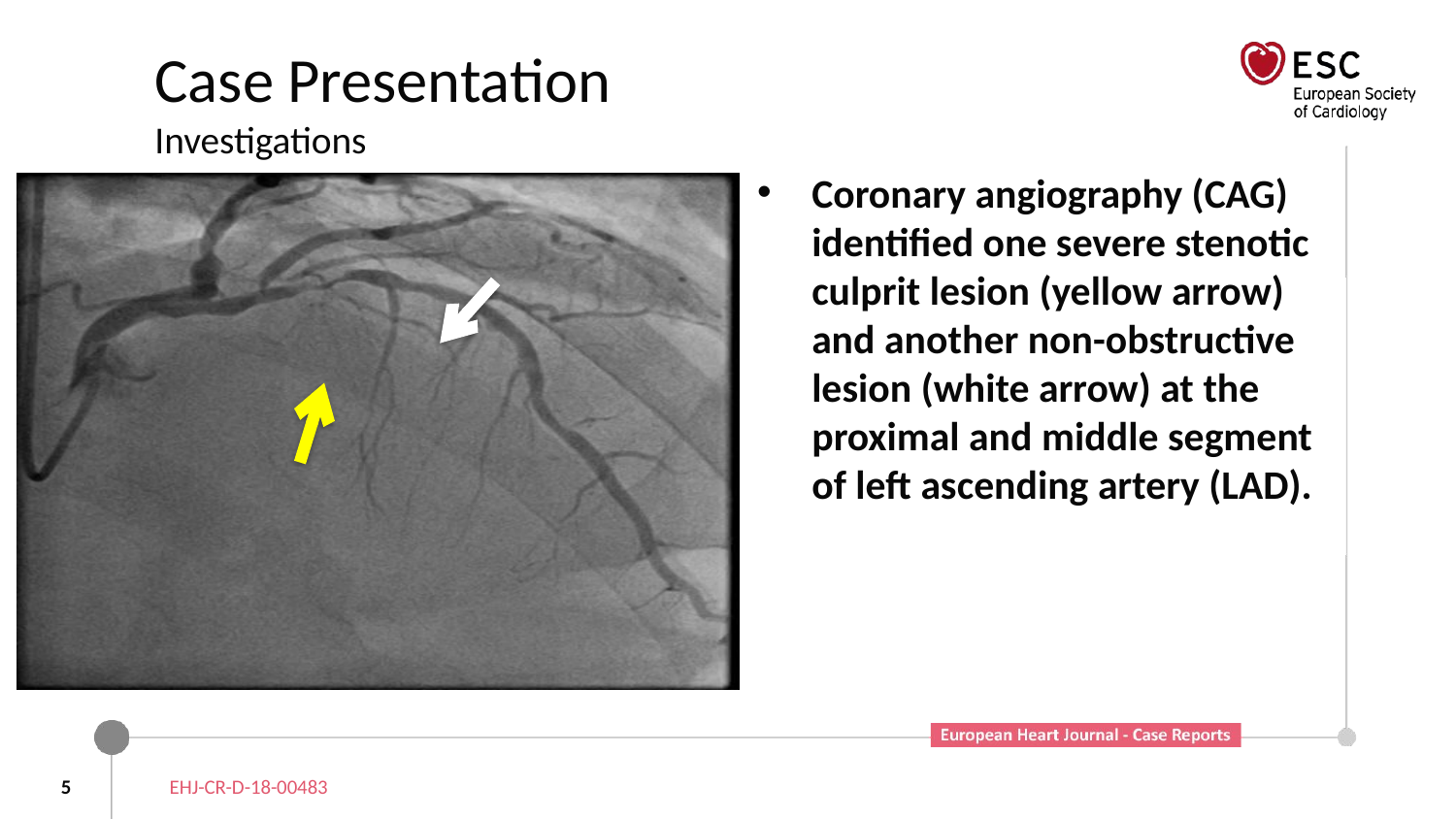

# Case Presentation Investigations
Coronary angiography (CAG) identified one severe stenotic culprit lesion (yellow arrow) and another non-obstructive lesion (white arrow) at the proximal and middle segment of left ascending artery (LAD).
5
EHJ-CR-D-18-00483

## Slide 6
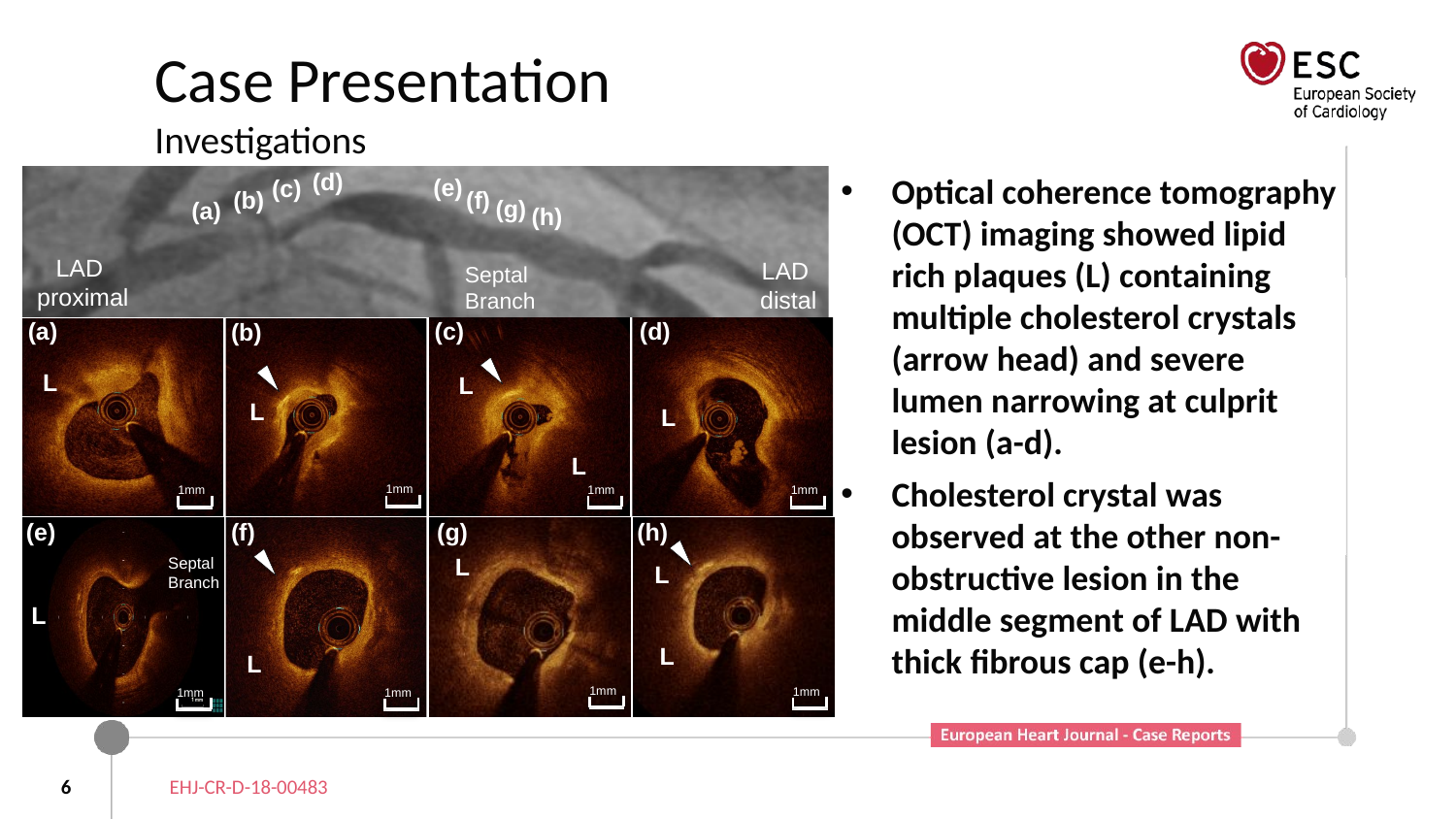

# Case Presentation Investigations
(d)
(e)
(c)
(b)
(f)
(g)
(a)
(h)
LAD
proximal
LAD
distal
Septal
Branch
a
b
Optical coherence tomography (OCT) imaging showed lipid rich plaques (L) containing multiple cholesterol crystals (arrow head) and severe lumen narrowing at culprit lesion (a-d).
Cholesterol crystal was observed at the other non-obstructive lesion in the middle segment of LAD with thick fibrous cap (e-h).
(c)
(d)
(a)
(b)
L
L
L
L
L
1mm
1mm
1mm
1mm
(g)
(h)
(f)
(e)
L
Septal
Branch
L
L
L
L
1mm
1mm
1mm
1mm
6
EHJ-CR-D-18-00483

## Slide 7
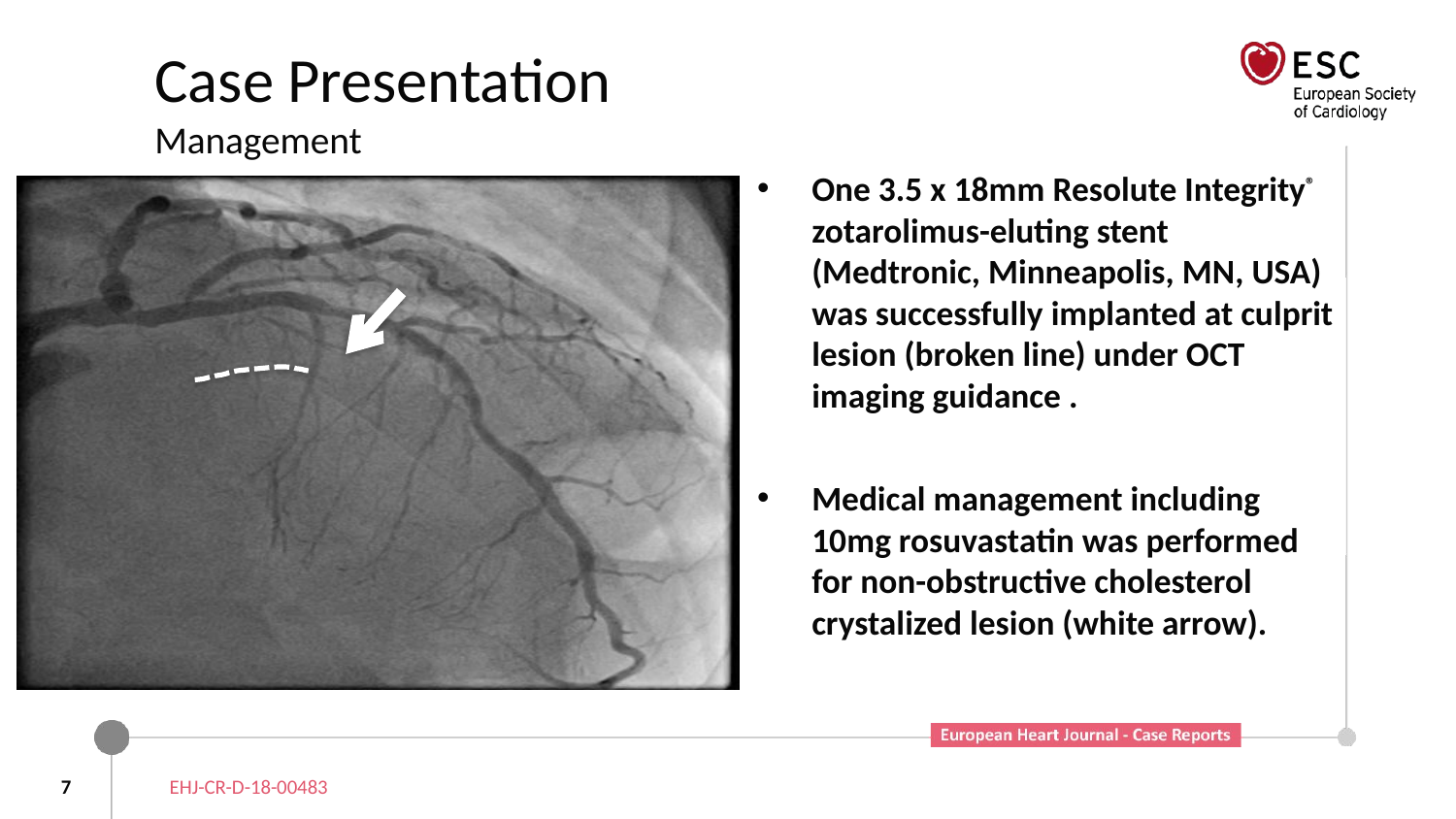

# Case Presentation Management
One 3.5 x 18mm Resolute Integrity® zotarolimus-eluting stent (Medtronic, Minneapolis, MN, USA) was successfully implanted at culprit lesion (broken line) under OCT imaging guidance .
Medical management including 10mg rosuvastatin was performed for non-obstructive cholesterol crystalized lesion (white arrow).
7
EHJ-CR-D-18-00483

## Slide 8
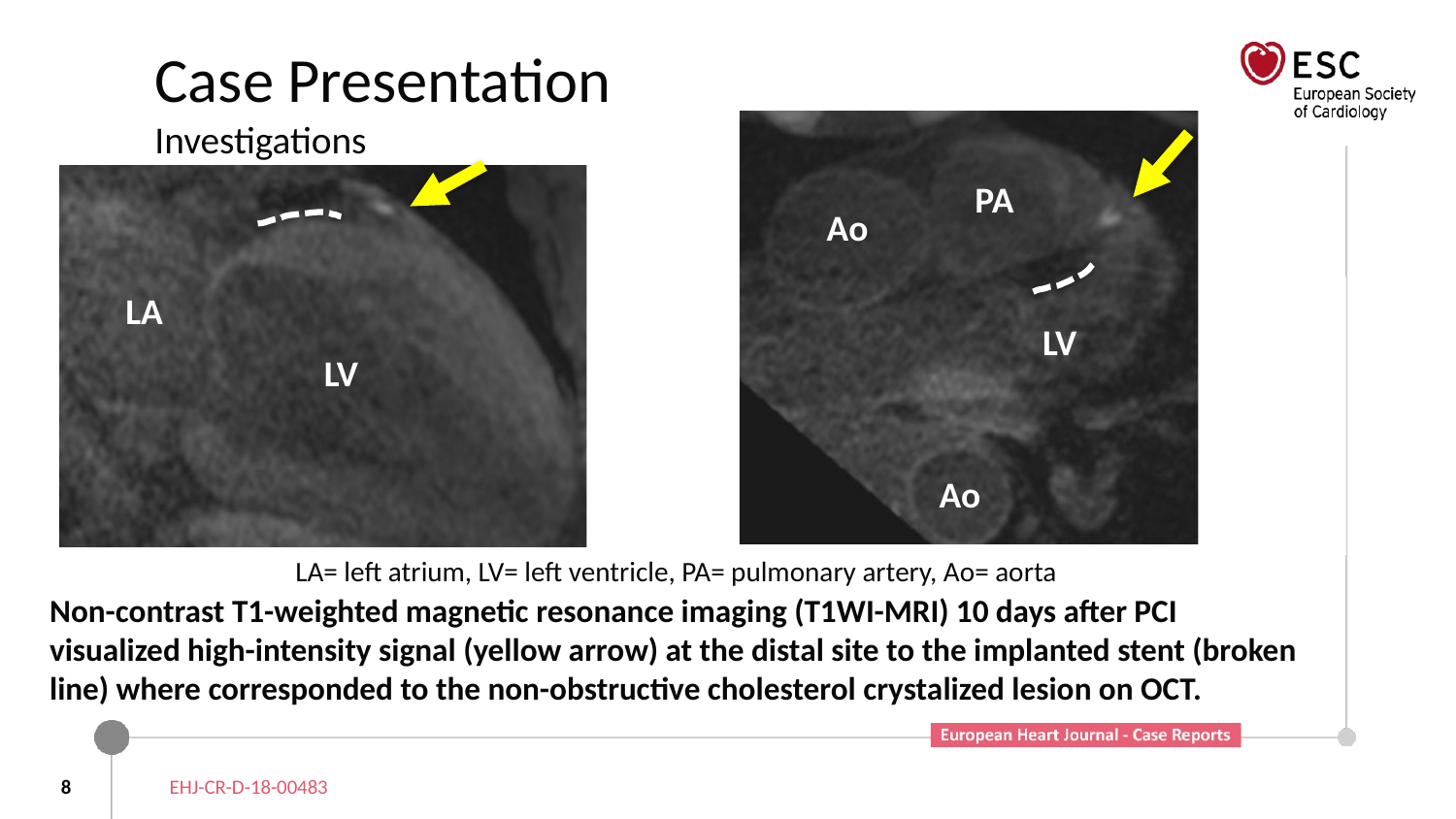

# Case Presentation Investigations
PA
Ao
LA
LV
LV
Ao
LA= left atrium, LV= left ventricle, PA= pulmonary artery, Ao= aorta
Non-contrast T1-weighted magnetic resonance imaging (T1WI-MRI) 10 days after PCI visualized high-intensity signal (yellow arrow) at the distal site to the implanted stent (broken line) where corresponded to the non-obstructive cholesterol crystalized lesion on OCT.
8
EHJ-CR-D-18-00483

## Slide 9
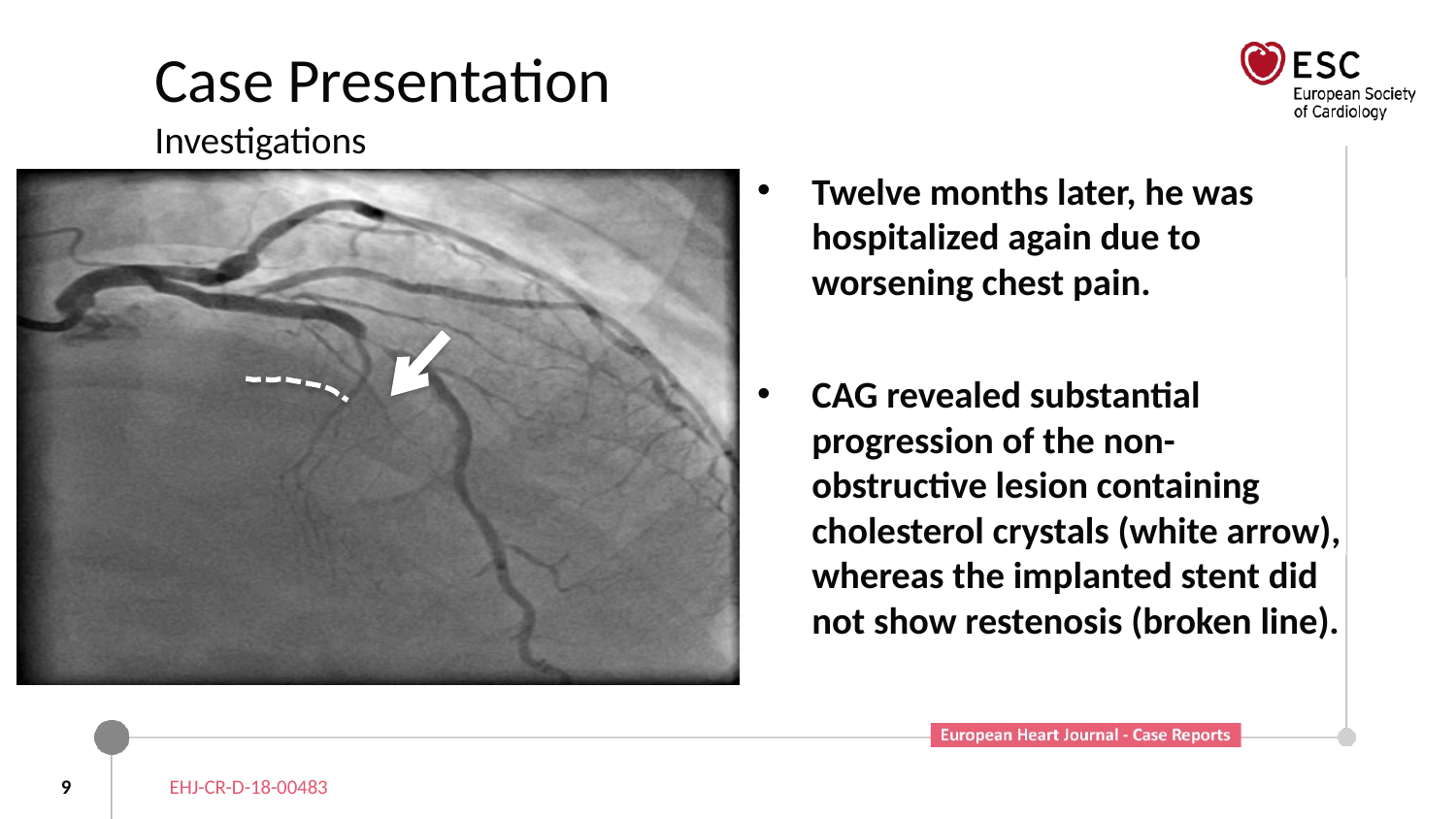

# Case Presentation Investigations
Twelve months later, he was hospitalized again due to worsening chest pain.
CAG revealed substantial progression of the non-obstructive lesion containing cholesterol crystals (white arrow), whereas the implanted stent did not show restenosis (broken line).
9
EHJ-CR-D-18-00483

## Slide 10
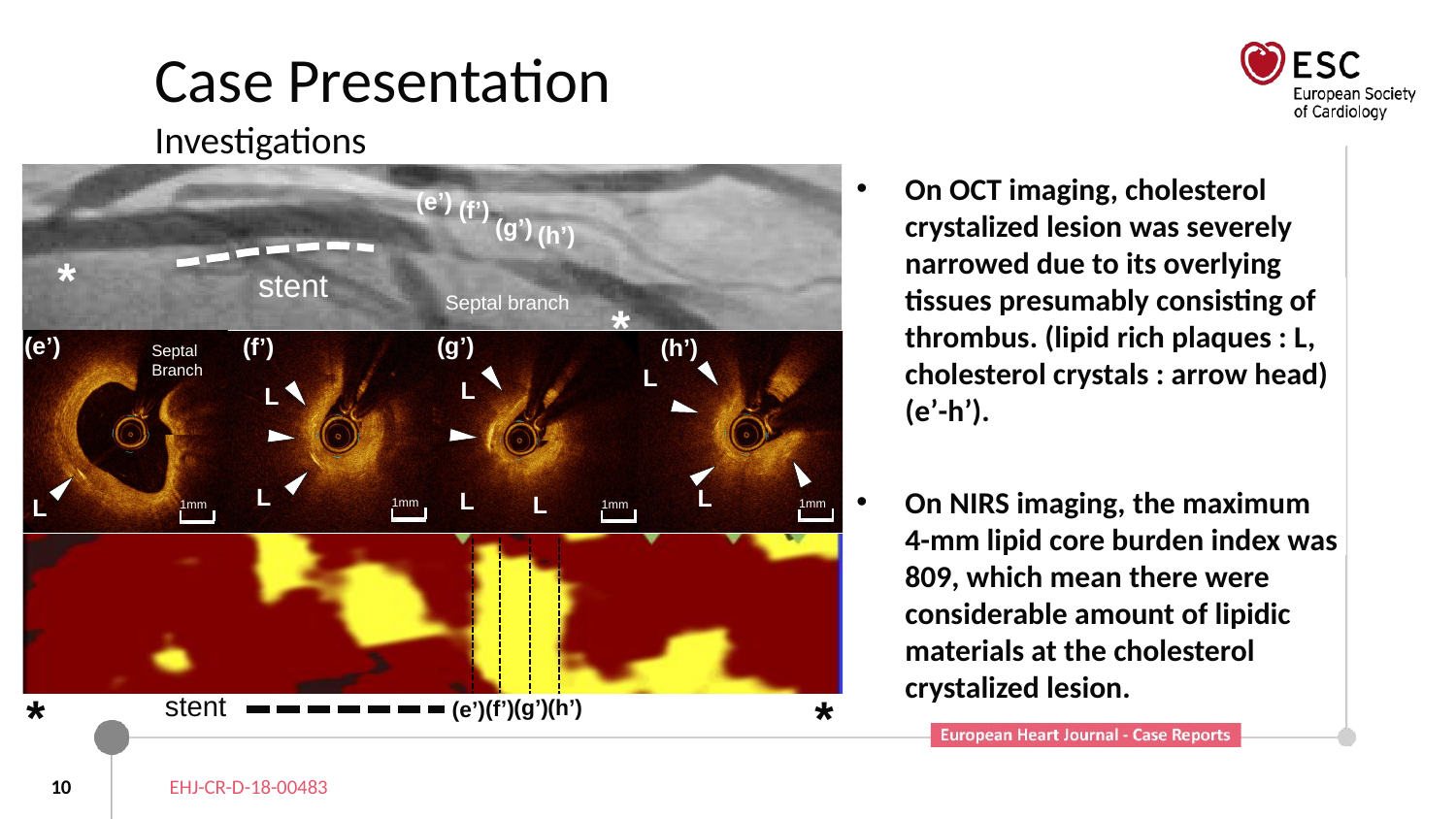

# Case Presentation Investigations
On OCT imaging, cholesterol crystalized lesion was severely narrowed due to its overlying tissues presumably consisting of thrombus. (lipid rich plaques : L, cholesterol crystals : arrow head) (e’-h’).
On NIRS imaging, the maximum 4-mm lipid core burden index was 809, which mean there were considerable amount of lipidic materials at the cholesterol crystalized lesion.
(e’)
(f’)
(g’)
(h’)
*
stent
Septal branch
*
(e’)
(g’)
(c)
(d)
(a)
(f’)
(b)
(h’)
Septal
Branch
L
L
L
L
L
L
L
L
L
L
L
L
L
1mm
1mm
1mm
1mm
(g)
L
L
stent
*
*
(g’)
(h’)
(f’)
(e’)
10
EHJ-CR-D-18-00483

## Slide 11
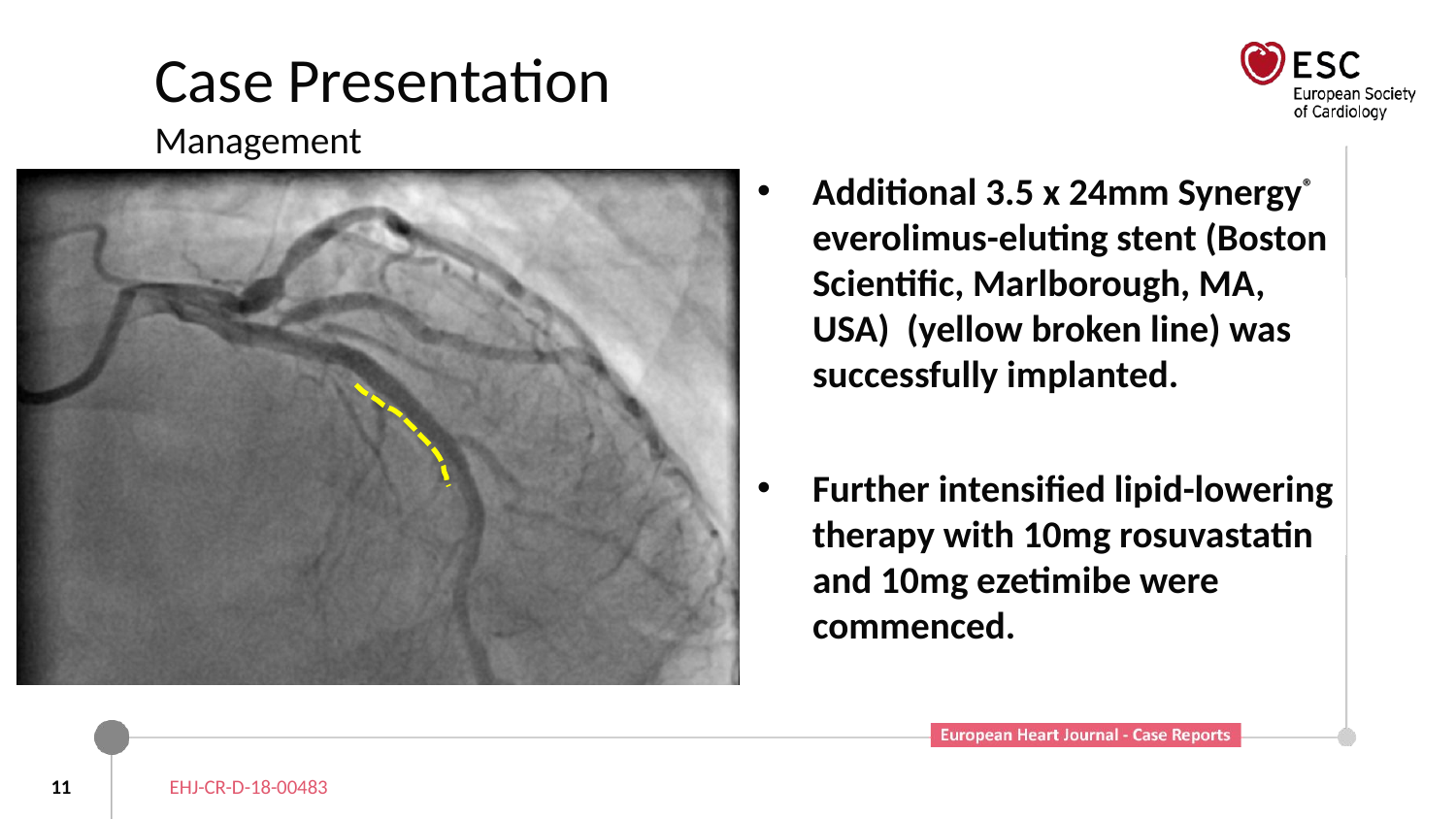

# Case Presentation Management
Additional 3.5 x 24mm Synergy® everolimus-eluting stent (Boston Scientific, Marlborough, MA, USA) (yellow broken line) was successfully implanted.
Further intensified lipid-lowering therapy with 10mg rosuvastatin and 10mg ezetimibe were commenced.
11
EHJ-CR-D-18-00483

## Slide 12
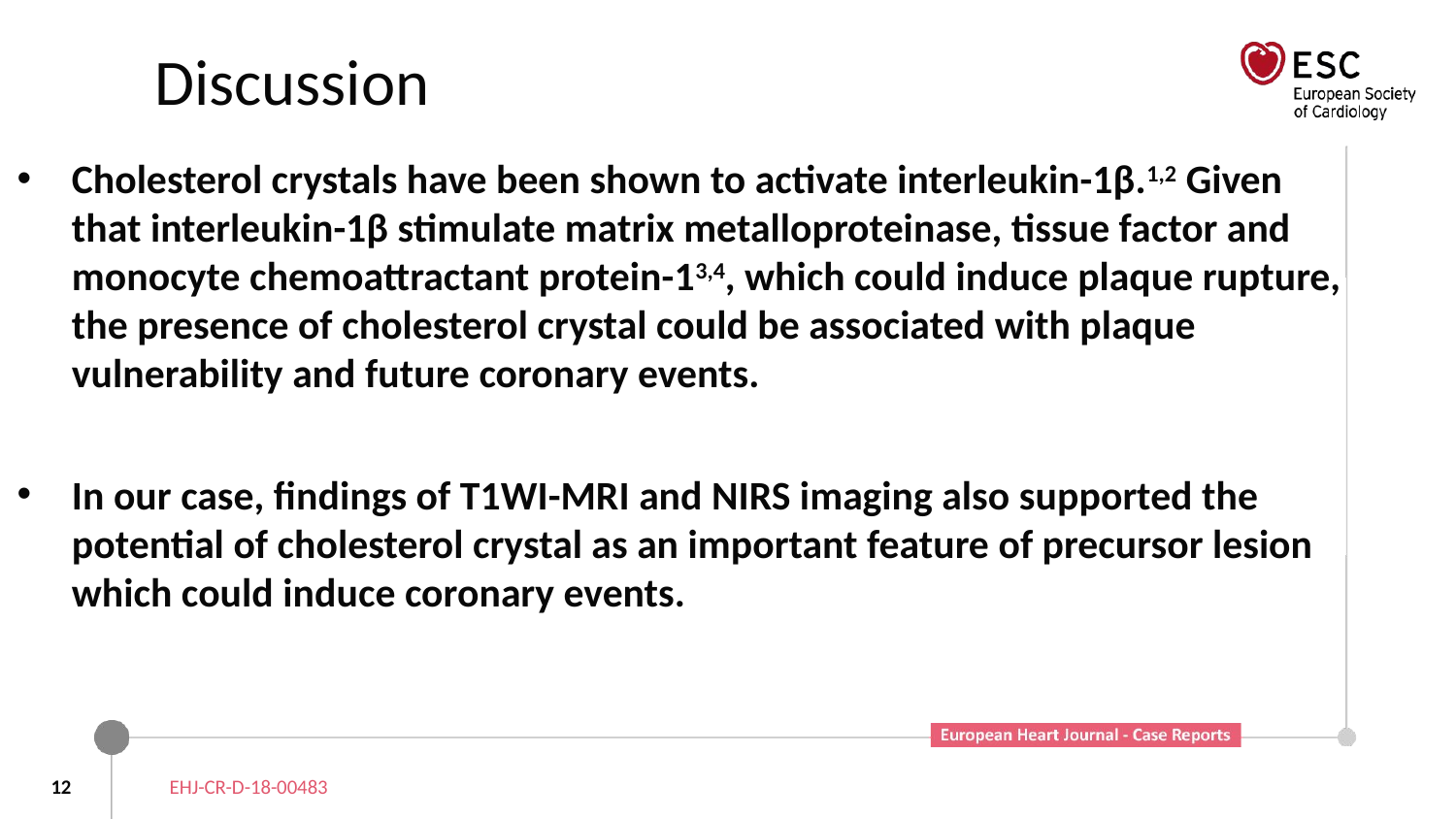

# Discussion
Cholesterol crystals have been shown to activate interleukin-1β.1,2 Given that interleukin-1β stimulate matrix metalloproteinase, tissue factor and monocyte chemoattractant protein-13,4, which could induce plaque rupture, the presence of cholesterol crystal could be associated with plaque vulnerability and future coronary events.
In our case, findings of T1WI-MRI and NIRS imaging also supported the potential of cholesterol crystal as an important feature of precursor lesion which could induce coronary events.
12
EHJ-CR-D-18-00483

## Slide 13
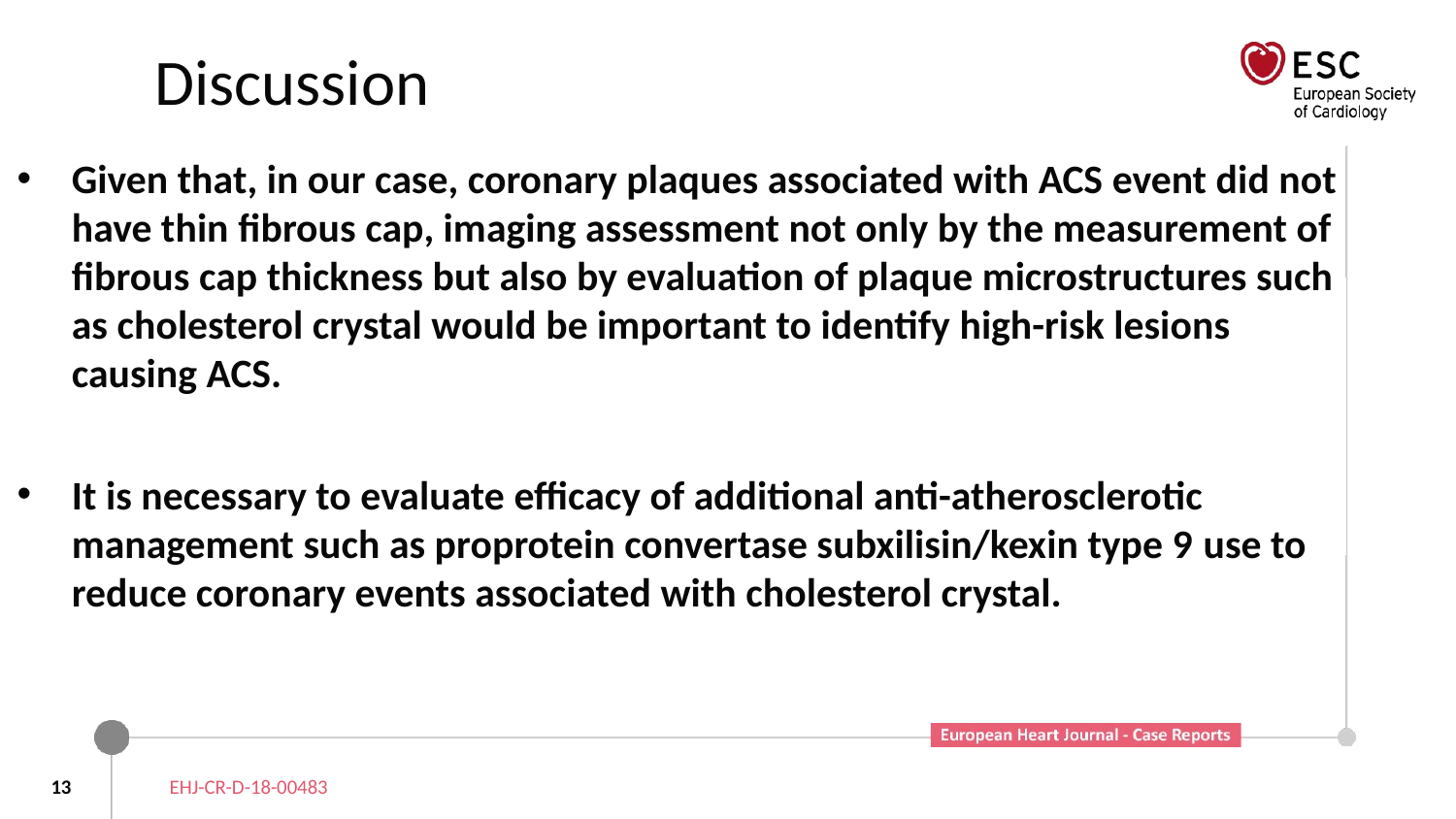

# Discussion
Given that, in our case, coronary plaques associated with ACS event did not have thin fibrous cap, imaging assessment not only by the measurement of fibrous cap thickness but also by evaluation of plaque microstructures such as cholesterol crystal would be important to identify high-risk lesions causing ACS.
It is necessary to evaluate efficacy of additional anti-atherosclerotic management such as proprotein convertase subxilisin/kexin type 9 use to reduce coronary events associated with cholesterol crystal.
13
EHJ-CR-D-18-00483

## Slide 14
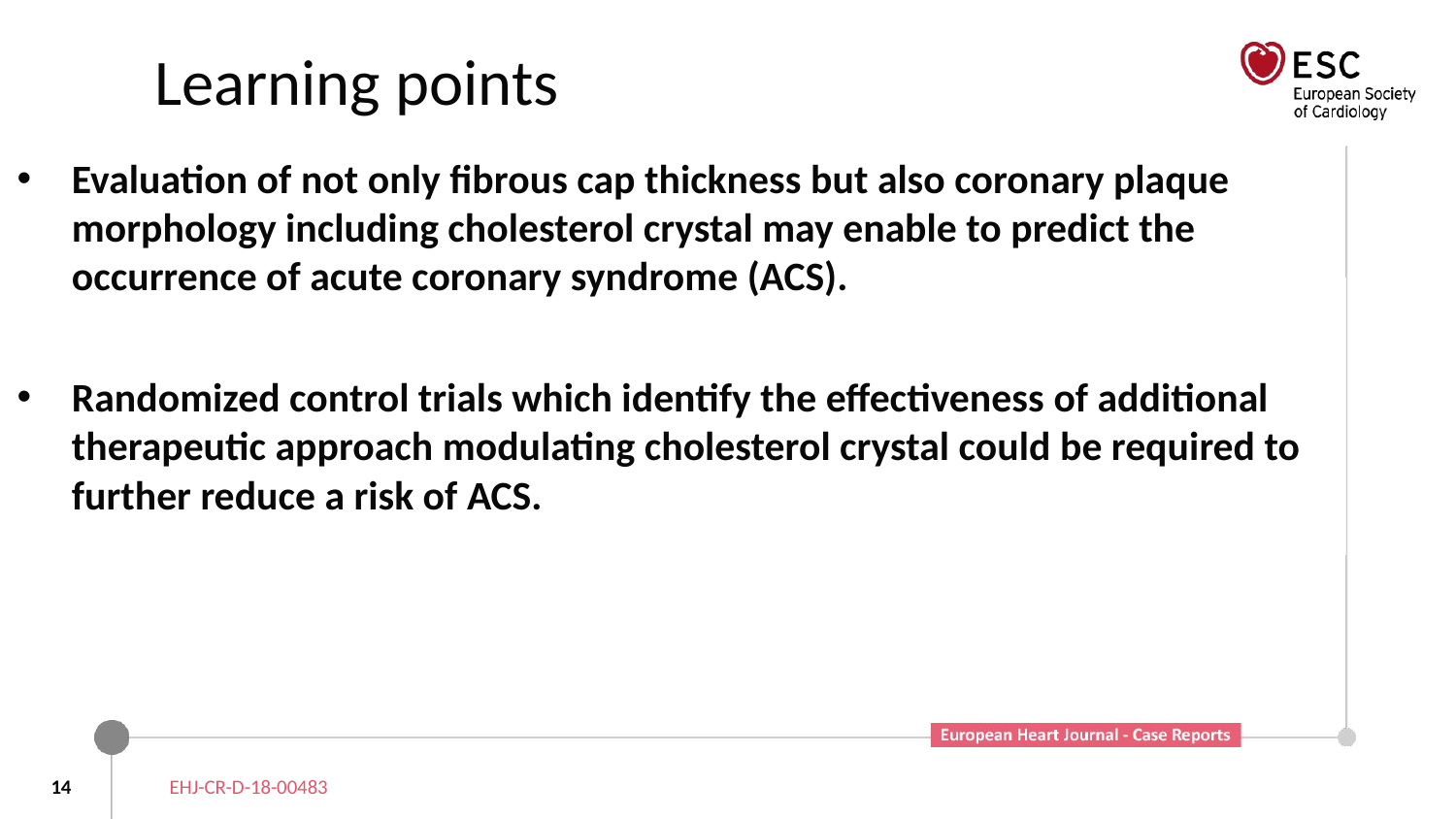

# Learning points
Evaluation of not only fibrous cap thickness but also coronary plaque morphology including cholesterol crystal may enable to predict the occurrence of acute coronary syndrome (ACS).
Randomized control trials which identify the effectiveness of additional therapeutic approach modulating cholesterol crystal could be required to further reduce a risk of ACS.
14
EHJ-CR-D-18-00483

## Slide 15
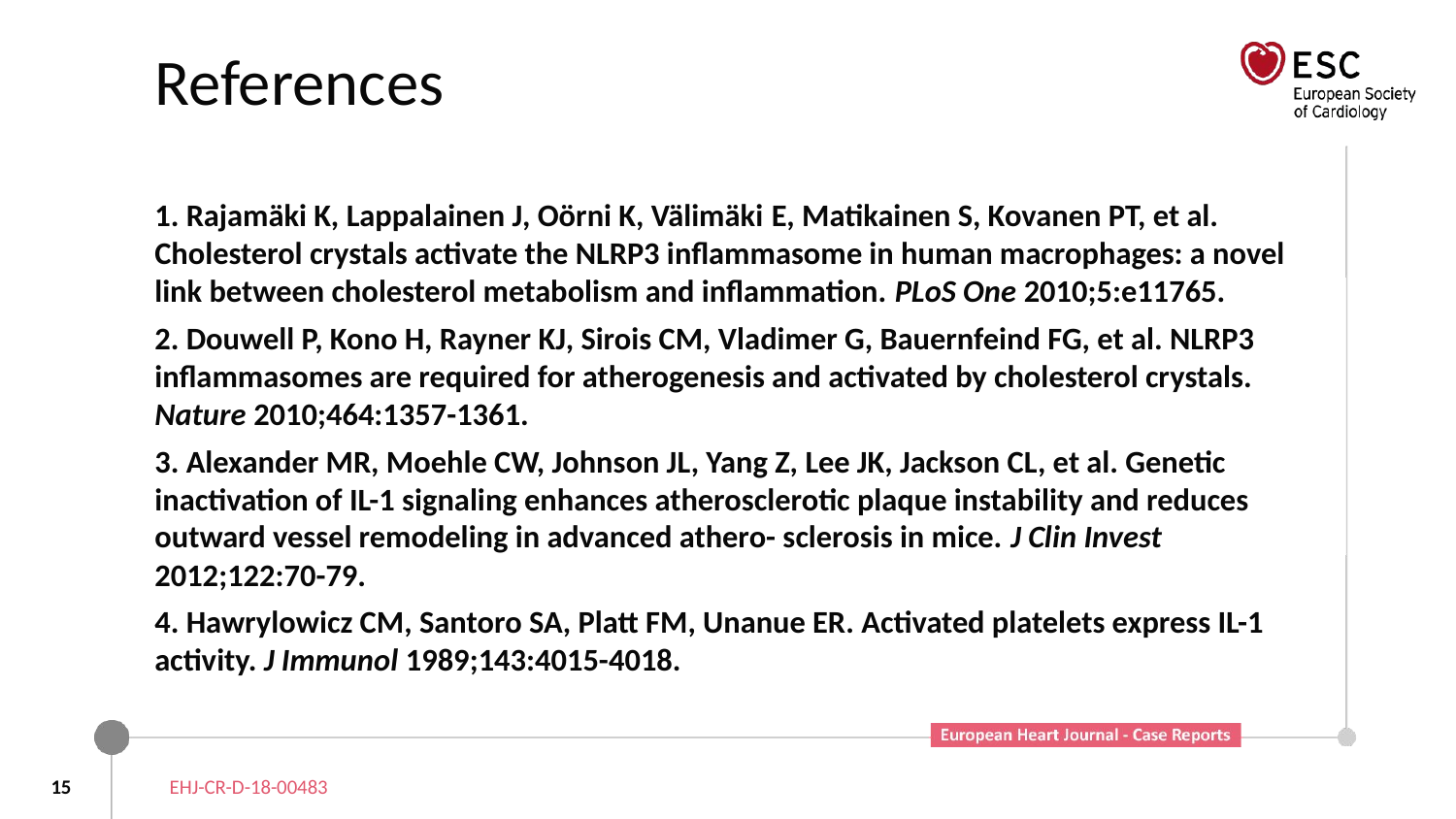

# References
1. Rajamäki K, Lappalainen J, Oörni K, Välimäki E, Matikainen S, Kovanen PT, et al. Cholesterol crystals activate the NLRP3 inflammasome in human macrophages: a novel link between cholesterol metabolism and inflammation. PLoS One 2010;5:e11765.
2. Douwell P, Kono H, Rayner KJ, Sirois CM, Vladimer G, Bauernfeind FG, et al. NLRP3 inflammasomes are required for atherogenesis and activated by cholesterol crystals. Nature 2010;464:1357-1361.
3. Alexander MR, Moehle CW, Johnson JL, Yang Z, Lee JK, Jackson CL, et al. Genetic inactivation of IL-1 signaling enhances atherosclerotic plaque instability and reduces outward vessel remodeling in advanced athero- sclerosis in mice. J Clin Invest 2012;122:70-79.
4. Hawrylowicz CM, Santoro SA, Platt FM, Unanue ER. Activated platelets express IL-1 activity. J Immunol 1989;143:4015-4018.
15
EHJ-CR-D-18-00483
